# Continuous Rural-Urban Coding for Cancer Disparity Studies: Is It Appropriate for Statistical Analysis?

**DOI:** 10.3390/ijerph16061076

**Published:** 2019-03-26

**Authors:** Lusine Yaghjyan, Christopher R. Cogle, Guangran Deng, Jue Yang, Pauline Jackson, Nancy Hardt, Jaclyn Hall, Liang Mao

**Affiliations:** 1 Department of Epidemiology, College of Public Health and Health Professions and College of Medicine, University of Florida, Gainesville, FL 32601, USA; lyaghjyan@ufl.edu; 2 Division of Hematology and Oncology, Department of Medicine, College of Medicine, University of Florida, Gainesville, FL 32601, USA; christopher.cogle@medicine.ufl.edu (C.R.C.); jackpa@ufl.edu (P.J.); 3 Department of Geography, College of Liberal Arts and Sciences, University of Florida, Gainesville, FL 32601, USA; guangrandeng@ufl.edu (G.D.); jy18007@uga.edu (J.Y.); 4 College of Medicine, University of Florida, Gainesville, FL 32601, USA; hardt@ufl.edu; 5 Institute for Child Health Policy, College of Medicine, University of Florida, Gainesville, FL 32601, USA; jaclynha@ufl.edu

**Keywords:** late stage cancer, rural-urban codes, continuous variable, health disparities

## Abstract

*Background*: The dichotomization or categorization of rural-urban codes, as nominal variables, is a prevailing paradigm in cancer disparity studies. The paradigm represents continuous rural-urban transition as discrete groups, which results in a loss of ordering information and landscape continuum, and thus may contribute to mixed findings in the literature. Few studies have examined the validity of using rural-urban codes as continuous variables in the same analysis. *Methods*: We geocoded cancer cases in north central Florida between 2005 and 2010 collected by Florida Cancer Data System. Using a linear hierarchical model, we regressed the occurrence of late stage cancer (including breast, colorectal, hematological, lung, and prostate cancer) on the rural-urban codes as continuous variables. To validate, the results were compared to those from using a truly continuous rurality data of the same study region. *Results*: In term of associations with late-stage cancer risk, the regression analysis showed that the use of rural-urban codes as continuous variables produces consistent outcomes with those from the truly continuous rurality for all types of cancer. Particularly, the rural-urban codes at the census tract level yield the closest estimation and are recommended to use when the continuous rurality data is not available. *Conclusions*: Methodologically, it is valid to treat rural-urban codes directly as continuous variables in cancer studies, in addition to converting them into categories. This proposed continuous-variable method offers researchers more flexibility in their choice of analytic methods and preserves the information in the ordering. It can better inform how cancer risk varies, degree by degree, over a finer spectrum of rural-urban landscape.

## 1. Introduction

The evidence for rural-urban disparities in late-stage cancer risk remains inconsistent [1,2]. In the current literature, many studies have reported a disproportionally higher risk of late-stage cancer among rural residents than their urban counterparts [3,4,5], which was hypothesized to result from geographical barriers in accessing cancer screening and treatment services, including long travel times and lack of providers. On the other hand, several recent studies support a hypothesis of ‘rural reversal’, arguing that the late-stage cancer risk for urban residents is greater than or equal to that for rural populations [6,7,8]. Since these studies adopted different methods to define rural-urban residence (Table 1), the current debates on residential disparities call for a re-consideration of how to represent rural-urban landscape.

Cancer researchers tend to rely upon rural-urban coding systems developed by government agencies (Table 1). Many of these systems use integer codes to represent rural-urban transition as a ‘continuum’, such as the Rural-Urban Continuum Codes (RUCC from 1 to 9) for counties [23], and the Rural-Urban Commuting Area (RUCA from 1 to 10) codes for census tracts and ZIP-code tabulation areas (ZCTA) [24,25], both developed by the US Department of Agriculture (USDA). Codes with low values (e.g., 1) indicate highly urbanized areas, while codes with great values (e.g., 10) represent highly rural areas. In a majority of cancer disparity studies, these integer rural-urban codes are further grouped into nominal variables to ease statistical analysis as well as interpretation (Table 1). For example, the RUCC codes are often dichotomized into ‘rural’ and ‘urban’ groups [4,12,13,14,16], and the RUCA codes are commonly classified into a few tiers, such as ‘urban cores’, ‘large rural towns’, and ‘small rural towns, and ‘isolated areas’ [11,18,19,26]. The conversion to nominal groups requires an assumption that cancer risk follows a step function where the risk within groups is homogeneous, leading to power loss and inaccurate estimation [27]. Moreover, the nominal groups are often treated as dummy variables in statistical analysis and the order between groups are not explicitly represented, which prevents researchers from investigating if ordering in rural-urban transition is a part of cancer risk. As pointed out by Hall, et al. [28] and Cossman et al. [29], the categorization of rural-urban codes may mask variability within the continuum and produce unstable analysis results. When health policy decisions are made based on such categorizations, inappropriate policy choices may result, e.g., low payments to counties with relatively high needs [29]. Both studies call for alternative rural-urban classifications or analytic methods. 

Instead of categorization, researchers may ignore the fact that those integer rural-urban codes are not really numeric, and treat them directly as a continuous variable in statistical analysis. As a premise, the rural-urban transition can be conceptualized as a continuous gradient, which is widely recognized in landscape ecology, economics, and regional geography [30,31]. It is therefore appropriate to model the rural-urban continuum as a gradually changing continuous variable, rather than ‘urban-suburban-rural’ categories. The use of integer codes as a continuous variable offers researchers more flexibility in their choice of analytic methods and preserves the information in the ordering. More importantly, it allows many researchers to analyze the data using techniques that their audience is familiar with and can easily understand, e.g., simple linear regression. The argument is that even if results are approximations, they’re understandable approximations. To the best of our knowledge, we only found one attempt to use RUCA as continuous variables in health-related analysis [32], but not in the cancer literature. For the continuous-variable method, there is an underlying assumption that the numerical interval between subsequent codes is equal. In other words, the magnitude of landscape variation between rural-urban code 1 and 2 should be the same as that between code 2 and 3. The validity of this equal-interval assumption has not been tested in the cancer literature, which may explain why no such studies have been seen in the literature. 

Taking advantage of recently published data regarding continuous rurality in Florida, we attempted to: (1) examine the appropriateness of using integer rural-urban codes as a continuous variable for analyzing cancer risk, and (2) suggest the optimal alternative when continuous rurality data is not available. To implement the proposed method, we took rural-urban disparities in late-stage cancer risk in north Florida as a case study. The research results are expected to provide a new alternative method to cancer researchers on representing rural-urban residence in their disparity studies.

## 2. Methods

### 2.1. Study Population

**Figure 1:**
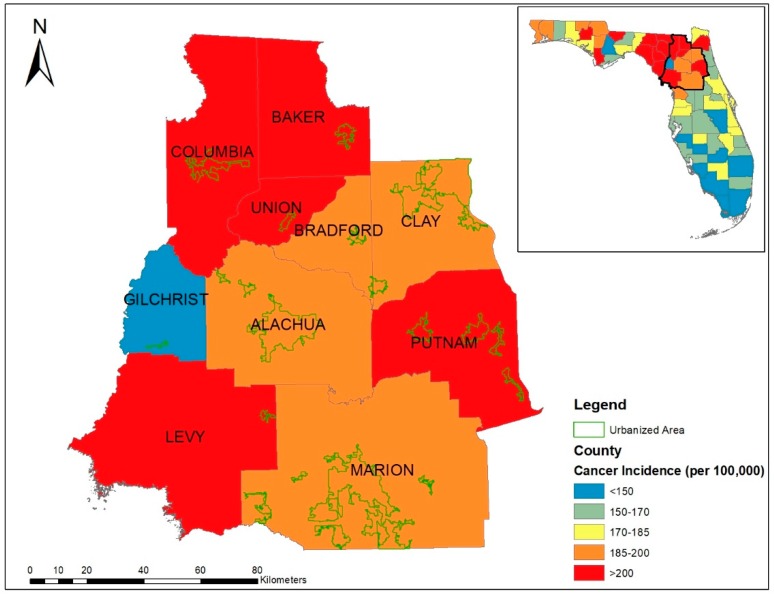
Age adjusted cancer incidence rates (at all cancer sites) for 10 counties in north Florida during 2008, derived from Florida department of Health. The inset map shows the spatial location and scope of study area in Florida.

The study area included 10 counties in the north central Florida with a total population of 1,040,304 (Figure 1). A mixture of rural to urban landscape makes it ideal for examining rural-urban differences in this population. According to cancer registry statistics of 2008, both age-adjusted cancer incidence and mortality rates by county (Figure 1) in the study area were significantly higher than in the rest of the state [33]. Therefore, health planning efforts have been directed towards addressing this health disparity concern. 

### 2.2. Cancer Registry Data

Information on cancer cases between year 2005 and 2010 were obtained from the population-based statewide cancer registry, the Florida Cancer Data System (FCDS). The FCDS is a joint effort of the Florida Department of Health and Miller School of Medicine at the University of Miami. It collects information on residential address, demographics, diagnosis, stage, medical history, laboratory data, tissue diagnosis, and initial course of treatment from every cancer patient in any hospital and outpatient facility licensed in Florida.

A total of 24,796 patient records were first geocoded based on the latitude and longitude of home addresses. Individuals with missing home addresses, race and gender information were excluded (6.5%), resulting in 23,283 geocoded cancer cases. According to the Surveillance, Epidemiology, and End Results (SEER) summary stage characterization, the cancer stage was reported using one of the six primary categories: in situ, localized, regional, distant, not applicable, and unstaged [34]. The ‘Not applicable’ and ‘Unstaged’ cases (*n* = 1179 or 5.1%) were not informative and thus excluded from the study, resulting a final study population of 22,104 cases. If an individual was diagnosed with more than one cancer, only the first diagnosis was used in the analysis. We further focused on the following major cancer sites that were defined using ICD-9 codes (See Table A1 in Appendix A for details): female breast (*n* = 5301), colorectal (*n* = 3005), hematological (*n* = 3100), lung (*n* = 5702), and prostate (*n* = 4996). Same as many previous studies [9,19,21,35], these cancer cases were dichotomized by their stage at diagnosis. The late stage cancers were defined as those with ‘regional’ and ‘distant’ metastasis and the early stage cancers included ‘in-situ’ and ‘localized’. The distribution for each cancer site by early and late stage is presented in Table 2. This study was approved by the Florida Department of Health and the University of Florida Institutional Review Boards (IRB201601383). 

### 2.3. Rural-Urban Codes

We considered four different types of rural-urban definitions at the county, ZCTA, census tract, and grid cell levels, respectively. The first three are derived from conventional integer coding systems, while the last one is from a real-number based continuous surface. 

For the county level, we used the RUCC system developed by USDA based on population size and closeness to urbanized or metropolitan areas. This system codes rural-urban landscape from number 1 to 9, with 1 indicating counties in metro areas of 1 million population or more, and 9 indicating completely rural or less than 2500 urban population, not adjacent to a metro area. 

For the ZCTA and census tract levels, we adopted the RUCA codes that are defined for census tracts based on population density, proximity to urban areas, and daily commuting patterns [25]. RUCA uses whole numbers from 1 to 10 to represent transition from metropolitan area core (1), to micropolitan area core (4), to small town core (7), and finally to rural area (10). Because many health datasets are collected at the ZCTA level, the ZIP code approximation of the census tract-based RUCA codes was also developed by rural health research center at the University of Washington [36].

For the grid cell level, we used a recently published cell grid of rurality for Florida at a spatial resolution of 600 m × 600 m, which is the average size of census blocks in the state [37]. Different from integer coding systems, there have been several efforts to develop and promote continuous rurality indices [37,38,39,40,41]. These works assume that the rural-urban gradient can be modeled as continuous surface, as a combined result from local demographic (population and ethnic diversity), socio-economic (household income and land use), and infrastructural measures (road network, access to health facilities and social services). Every cell location, thus, contains a real-number rurality index ranging from 0 (the most urban) to 10.00 (the most rural). More information about factor selection for Florida rurality map [37], and the supporting literature can be referred to Table A2 in Appendix A.

### 2.4. Statistical Analyses with Rural-Urban Codes as a Continuous Variable

To test the appropriateness of using integer rural-urban codes as a continuous variable, we adopted a two-level logistic regression model to explore the associations between rural-urban residence and late-stage cancer risk. The two levels considered individual patients being nested within a smaller number of geographic areas. The cancer stage (early or late) of individual patients (defined in Section 2.2) was the dependent variable. At the patient level, the age at diagnosis, race, and gender (except female breast cancer and male prostate cancer) were included as covariates. At the geographic level (county, ZCTA, or census tract), we considered the integer rural-urban codes at the relevant level as a continuous variable (rather than a nominal variable). The risk was estimated as odds ratios (ORs) and corresponding 95% confidence intervals. The findings were then compared against the results of same analysis with the cell-based continuous rurality index, which was considered as the best available reference in the study area (justified in Section 3.1). If it is valid to use integer rural-urban codes as a continuous variable, the results are expected to similar. 

### 2.5. Optimal Rural-Urban Codes as a Continuous Variable

Although integer rural-urban coding systems cover the entire U.S., the continuous rurality grid is only found available in Florida so far. A question of interest is: if the continuous rurality grid is not available, which integer coding system is the optimal alternative to be used as a continuous variable? In other words, which existing rural-urban coding system offers the closest results as the continuous rurality grid does? To answer this question, we calculated the root mean squared error (RMSE) of ORs (obtained from Section 2.4) for each integer coding system. The ‘error’ was the difference between the OR of an integer coding system and the OR of continuous rurality index, and was calculated for each of the five cancer sites. A lower RMSE indicates that the integer rural-urban coding system produces a closer estimation to that from the continuous rurality index.

## 3. Results and Discussion

### 3.1. Comparison of Rural-Urban Codes

**Figure 2:**
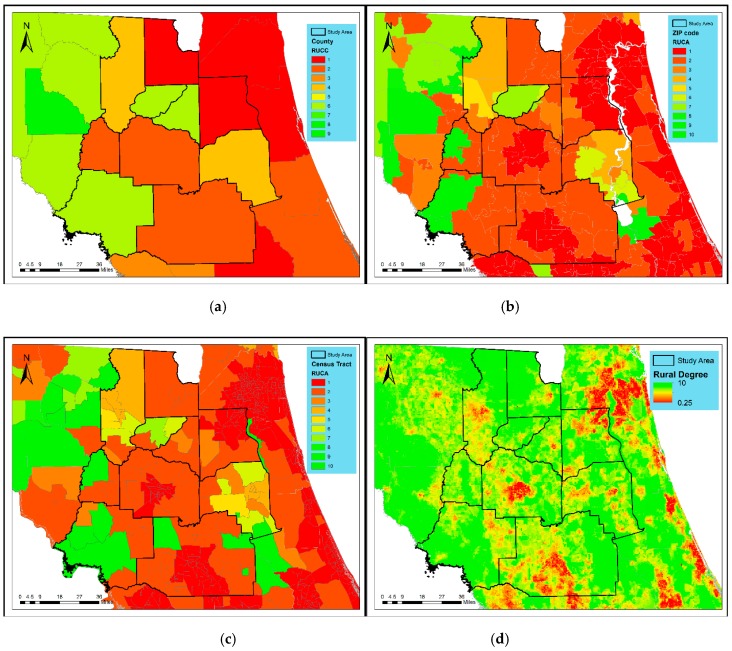
Rural-urban classifications at 4 different spatial levels: (**a**) the RUCC for counties, (**b**) the RUCA coding system for ZIP code tabulation areas (ZCTA), (**c**) the RUCA for census tracts, and (**d**) the cell-based continuous rurality index. For all, greater values indicate higher degrees of rurality.

Among the four rural-urban definitions, the county-level RUCCs (Figure 2a) characterize the most homogenous rural-urban landscape, in that residents’ rural exposure are all identical in the same county. This definition is susceptible to biases of averaging and misclassification as rural residents living in a county that is only partly urban are assigned an urban code. The RUCA for ZCTAs and census tracts (Figure 2b,c) offers finer granularity of rural-urban transition than the RUCC does. For those small ZCTAs or census tracts, the assumption of homogeneity may stand. However, for a large ZCTA or census tract (most likely in suburban and remote areas), a single RUCA number may oversimplify the local rural-urban variation. 

Compared with RUCC and RUCA, the 600 m cell-based rurality map (Figure 2d) breaks rigid statistical boundaries, and thus exhibited a smoother transition from highly developed urban areas, to suburban areas, to small towns, and then very rural areas. The grid cells, with an average size similar to census blocks or street blocks, are the smallest representation of human habitats where rural-urban features can be reasonably assumed homogeneous.

The rurality index (Figure 2d) ranges from 0.60 to 10 (with a population weighted average of 5.24), indicating that the study region has a full range of rural-urban continuum, where the population is concentrated in suburban area. Table 3 shows the distribution of continuous rurality index in conventionally integer rural-urban codes. The population weighted mean of rurality index increases as the conventional rural-urban code increases, indicating some extent of agreement in defining urban-rural residence among different methods. The value ranges, however, imply that the homogeneity assumption made by conventional methods is questionable as there is a wide variation of rurality index within each code and overlap across classification-specific categories. Many cell locations with great values of rurality index can be misclassified into the urban class, and vice versa. For instance, Code 1 for RUCC and RUCA is defined for highly urbanized areas, but can misrepresent many sub-urban cell locations as demonstrated by the mean index value of 4.3–4.8 reflective of a mixed nature of suburban and urban areas. 

Table 3 also indicates the population distribution among discrete rural-urban codes, as well as one-degree intervals of continuous rurality. The RUCC and RUCA tend to classify a majority of population into code 1 and 2 (highly urbanized), while the continuous rurality index deems them as ‘suburban’ (value range from 3 to 6). Therefore, there is a clear pattern that people living in the suburban area are prone to be misclassified by discrete coding systems as residents in highly urbanized areas, because each census unit is assumed to be homogeneous.

The concept of ‘rural’ is complex, multifaceted, and often vague. Government agencies often define rurality based on one or two factors, for example, population size and adjacency to metro areas. However, many urban/rural studies argued that the rurality should be conceptualized in a more comprehensive way, and measured as a composite indicator [38,39,40]. As compared to conventional rural-urban codes, the continuous rurality index is not only consistent with the multi-facet ‘rural’ definition in the literature, but also offers a spatially resolved and continuous representation. The statistical analysis using continuous rurality index, as a continuous variable, could minimize the bias from ecological fallacy, and produce more valid associations. For all reasons above, we believe the continuous rurality index is sufficient to be used as the best available reference for subsequent comparison. 

### 3.2. Rural-Urban Disparities

Since all rural-urban codes were treated as continuous variables, their slopes in regression can be interpreted the same way as other widely used continuous variables, such as age, body weight, and temperature. In the multi-level regression analysis using RUCC at the county level (Table 4), we found weak but significant associations between RUCC and late-stage breast cancer (OR = 0.93; 95% Confidence Interval (CI) 0.88–0.99) and colorectal cancer (OR = 0.92; 95% CI 0.88–0.95), but no associations for other cancer sites. That is, one level increase in RUCC is associated with 7% decrease in the odds of late-stage breast cancer and 8% decrease in the odds of late-stage colorectal cancer. At the ZCTA level, we only identified a negative association between RUCA and late stage colorectal cancer (OR = 0.96; 95% CI: 0.92–0.99). The RUCA at the census tract level was not associated with late-stage cancer risk for any major cancer site. At the grid cell level, we identified a marginally significant decrease in the risk of late-stage colorectal cancer per each one degree increase in continuous rurality index (OR = 0.94; 95% CI 0.90–0.99). The slope can be interpreted as the odds of late stage diagnosis will decreases by 6% if the rurality index increases by one degree. No significant associations were found for other cancer sites.

The odd ratios across multiple spatial scales (Table 4) indicated a slight ‘rural reversal’ in late-stage colorectal cancer diagnoses in north central Florida, and this is consistent with some previous studies that reported a decrease in colorectal cancer risk living in rural areas [3,19]. We found no significant urban-rural disparities in late stage diagnosis for breast malignancies in women. This is inconsistent with prior studies of geographic disparities in breast cancer in Florida. For example, Amey et al. found a ’rural disadvantage’ [42], where participants were categorized as either ‘urban’, ‘adjacent rural’, or ‘nonadjacent rural’. In contrast, Mackinnon et al. reported an ‘urban disadvantage’ [12], where participants were classified as either ‘rural’ or ‘urban’. Despite of different time, such inconsistency can be attributed to different representations of rural-urban landscape (continuous vs. discrete) in the statistical analysis. For lung, prostate, or hematological cancer, the results consistently indicate that the rural-urban residence may have no effects on late-stage diagnosis.

Overall, Table 4 shows that the ORs for RUCC and RUCA codes slightly differ from those for the truly continuous rurality index. Such slight differences may be attributed to the modifiable areal unit problem (MAUP), where associations derived from data aggregated to a particular set of spatial units (cells) may change if one aggregates the same underlying data to a different set of units (counties, ZCTAs, and census tracts). However, the direction (positive or negative) and statistical significance of associations does not vary too much between different rural-urban codes, and most of them are in line with those suggested by the continuous rurality index. In short, we argue that the use of rural-urban codes at the county, ZCTA or census tract level as continuous variables could reach plausible answers, but the reliability remains uncertain unless a fine-scale cell-based analysis is performed. The advantage is that it allows researchers to look at how late stage cancer risk varies over a continuous rural-urban gradient, rather than a few of predefined categories. 

### 3.3. Optimal Rural-Urban Codes as a Continuous Variable

Table 5 shows that the RUCA for census tracts provided the lowest RMSE and thus the closest approximation to the outcomes from the continuous rurality index. The RUCC for counties may not be a good choice due to its coarse granularity over space, particularly for the Florida state where each county is large in size. The RUCA for ZCTAs is only an approximation of that for census tracts based on a crosswalk between census tracts and ZCTAs [24], and thus is not as reliable as the RUCA for census tracts. Overall, we would suggest using RUCA for census tracts as a continuous variable in cancer studies, if there is no spatially resolved continuous data available. 

Our study has a few limitations. First, 6.5% cancer cases were excluded from the analysis for missing information on home addresses, race and gender. Due to no locations, we have little knowledge on the spatial distribution of excluded cases, for example, rural vs. urban areas. Potential selection bias may be introduced from data imputation. Second, we have not tested the generalizability of our results outside the study area, as the rural-urban disparities in late stage cancer diagnosis have been documented to vary dramatically over geographic locations. The extended analysis can be easily performed when the continuous rurality index is developed for other regions. Third, like many other studies [14,35], we did not consider temporal variation of rural-urban landscape between 2005 and 2010, but assumed it was constant as that of 2010. Future studies would benefit from investigating how the rural-urban inequalities longitudinally [7]. Nevertheless, the government report of our study area showed that both demographics and socio-economy had a slow growth between 2005 and 2010 [43]. In this sense, our assumption of constant rural-urban continuum across the study area is reasonable.

## 4. Conclusions

The dichotomization or categorization of rural-urban codes is a prevailing paradigm in cancer disparity studies. The categories forcibly discretize rural-urban transition that is intrinsically continuous, and thus may contribute to the mixed associations found in the literature. In this study, we proposed using integer rural-urban codes as continuous variables, rather than nominal variables, in cancer disparity studies, and demonstrated its validity in the study of five major types of cancer in north central Florida. We argued that statistical analysis on continuous rural-urban variable could avoid implausible assumptions that cancer risk does not vary within categories, and thus better informs how the risk varies over a full spectrum of rural-urban landscape. Furthermore, the continuous-variable method offers researchers more flexibility in their choice of analytic methods and preserves the information in the ordering. Again, the focus of this study is the appropriateness of continuous-variable method in cancer studies. We do not attempt to argue that the continuous-variable method is superior to the conventional categorical-variable method, and vice versa. Both methods have their own strengths and weaknesses. The choice of methods depends on how much granularity a researcher needs to depict the rural-urban landscape and to interpret their results for policy making. 

Further, as financial resources for cancer prevention and control are limited, it is important to identify spatially resolve areas of increased cancer risk to guide cancer centers and departments of health in allocation of resources and interventions. The cell-based continuous rurality index is more appropriate to answer whether rural residence portends for a higher risk of late stage cancer. The fine-grained rurality map can help reveal high risk or high need areas at a census-block level, which would be otherwise masked by using conventional rural-urban codes by county, ZTCA, or tract. Such knowledge answers the emerging call for precise interventions, referred to as ‘the right intervention to the right population at the right time’ in order to maximize cost-effectiveness. For example, the spatially resolved risk map can be used to plan optimal transportation routes and stops for mobile cancer screening service within an extensive geographic region [44]. The mobile service does not have to stop by every census block, but only those with high risk, making the mobile strategy more feasible and effective but less costly. Likewise, another example would be an invitation strategy that sends advance notification letter to a population for cancer screening [45]. The postal mails can be prioritized to households only in those high need census blocks, rather than in the entire county or tract. In the circumstance of limited budget, the early notification letters can be sent to the maximal possible population who are most likely to need the screen service. Unfortunately, no such cell-based rural-urban classification is available across the United States. The research highlights an impressing need of developing fine grained rural-urban classifications by authorities, such as the federal and state agencies.

## Figures and Tables

**Table 1:** Cancer studies in the US with different rural-urban definitions and categorization.

Rural-Urban Definition	Study Areas and References	Cancer Site	Code Categorization	Analysis Unit	Disadvantage Reported
US Census Dichotomy	Florida [9]	Cervical *	Urban/Rural	Tract	No ^a^
	Mississippi [10]	All (incidence)	Urban/Rural	County	No
OMB definition	Nebraska [11]	Colorectal *	Rural/Mircopolitan/Metropolitan	County	No
USDA Rural-Urban Continuum Codes (RUCC)	Illinois [4]	Urological (mortality)	Urban (≤3)/Rural (4–9)	County	Rural
	Florida [12]	Breast *	Urban (≤3)/Rural (4–9)	Block group ^e^	Urban
	Entire US [3]	Colorectal, Lung *	Urban (≤3)/Rural (7 & 9)	County	Urban
	Georgia [13]	Breast *	Urban (≤3)/Rural (≥6)	County	Rural
	Missouri [14]	Breast *	Urban (≤3)/Rural (≥6)	County	Rural
	Georgia [15]	Colorectal *	Urban (≤3)/Rural (≥6)	County	No
USDA Rural-Urban Commuting Area (RUCA) codes	Pennsylvania [16]	Laryngeal *	Urban/Rural ^b^	ZCTA	Rural
	New Hampshire [8]	Breast *	Urban/Large rural town/Small rural town ^c^	ZCTA	No
	Georgia [17]	Colorectal *	Urban/Large rural town/Small rural town ^c^	Tract	Rural
	California [18]	Colorectal *	Urban/Large rural town/Small rural town ^c^	Tract	No
	Illinois [19]	Breast, Colorectal, Prostate, Lung *	Urban/Large rural town/Small rural town/Isolated rural ^d^	ZCTA	Urban
	Entire US [20]	Lung (mortality)	Urban/Large rural town/Small rural town/Isolated rural ^d^	Tract	No
	10 US states [21]	Breast *	Urban/Large rural town/Small rural town/Isolated rural ^d^	Tract	No

* Indicates studies on early or late cancer diagnosis; other studies are specified in parenthesis. ^a^: No significant differences between rural-urban categories. ^b^: RUCA’s 2 categories: Urban [1.0, 1.1, 2.0, 2.1, 3.0, 4.1, 5.1, 7.1, 8.1, 10.1]; Rural [4.0, 4.2, 5.0, 5.2, 6.0, 6.1, 7.0, 7.2, 7.3, 7.4, 8.0, 8.2, 8.3, 8.4, 9.0, 9.1, 9.2, 10.0, 10.2, 10.3, 10.4, 10.5, 10.6]. ^c^: RUCA’s 3 categories: Urban [1.0, 1.1, 2.0, 2.1, 3.0, 4.1, 5.1, 7.1, 8.1, 10.1]; large rural town [4.0, 4.2, 5.0, 5.2, 6.0, 6.1]; small rural town [7.0, 7.2, 7.3, 7.4, 8.0, 8.2, 8.3, 8.4, 9.0, 9.1, 9.2, 10.0, 10.2, 10.3, 10.4, 10.5, 10.6]. ^d^: RUCA’s 4 categories [22]: Urban [1.0, 1.1, 2.0, 2.1, 3.0, 4.1, 5.1, 7.1, 8.1, 10.1]; large rural town [4.0, 4.2, 5.0, 5.2, 6.0, 6.1]; small rural town [7.0, 7.2, 7.3, 7.4, 8.0, 8.2, 8.3, 8.4, 9.0, 9.1, 9.2]; isolated [10.0, 10.2, 10.3, 10.4, 10.5, 10.6]. ^e^: Each block group was assigned a RUCC from the county it is located in.

**Table 2:** Summary of cancer cases by stage and by primary site for analysis.

Stage	Breast (Female)	Colorectal	Hematological	Lung	Prostate
Early	3808	1318	190	1191	4310
Late	1493	1687	2910	4511	686
Total	5301	3005	3100	5702	4996

**Table 3:** Distribution of continuous rurality index in three conventionally used rural-urban codes.

RUCC-County ^a^			RUCA-ZCTA			RUCA-Tract			Continuous Rurality Index	
Discrete Code	Range of Rurality Index (Weighted Mean)	Population	Discrete Code	Range of Rurality Index (Weighted Mean)	Population	Discrete Code	Range of Rurality Index (Weighted Mean)	Population	Range	Population
1	0.60–9.87 (4.87)	21%	1	0.60–10 (4.58)	66%	1	0.60–9.99 (4.37)	57%	0–1	4%
2	0.70–9.93 (4.95)	58%	2	3.07–10 (6.92)	19%	2	3.07–9.96 (6.90)	25%	1–2	3%
3	-		3	3.08–9.96 (6.93)	3%	3	3.08–9.93 (7.15)	3%	2–3	5%
4	1.41–9.99 (6.17)	14%	4	1.41–10 (5.55)	5%	4	1.41–9.94 (5.21)	5%	3–4	13%
5	-		5	4.42–9.26 (6.88)	1%	5	4.87–9.86 (6.84)	1%	4–5	19%
6	2.64–9.92 (6.77)	8%	6	4.11–9.94 (6.55)	2%	6	4.63–9.92 (6.98)	2%	5–6	17%
7	-		7	2.64–9.80 (6.59)	2%	7	2.64–9.71 (6.04)	2%	6–7	16%
8	-		8	-		8	-		7–8	15%
9	-		9	-		9	-		8–9	7%
-	-		10	4.78–9.92 (7.38)	2%	10	4.78–10 (7.53)	4%	9–10	0%

**^a^** The RUCC by county is coded from 1 to 9, with greater codes indicating more rurality (same for all other classifications). The study area does not have counties with a RUCC code of 5, 7, 8, and 9.

**Table 4:** Associations between multiple classifications of rurality and the late-stage cancer (Odds ratios and 95% confidence interval).

Rural Definition	Breast	Colorectal	Hematological	Lung	Prostate
**RUCC-County**	0.934 ^a^	0.918	0.951	0.997	1.037
	(0.875, 0.998)	(0.884, 0.954)	(0.784, 1.153)	(0.969, 1.027)	(0.937, 1.147)
**RUCA-ZCTA**	0.970	0.956	1.046	1.004	1.041
	(0.932, 1.008)	(0.924, 0.988)	(0.971, 1.126)	(0.975, 1.033)	(0.975, 1.121)
**RUCA-Tract**	0.978	0.968	1.010	0.989	1.038
	(0.946, 1.010)	(0.935, 1.002)	(0.939, 1.087)	(0.958, 1.022)	(0.986, 1.092)
**Continuous rurality-Grid cell** ^b^	0.964	0.943	0.993	0.990	1.006
	(0.924, 1.005)	(0.900, 0.989)	(0.916, 1.077)	(0.945, 1.037)	(0.952, 1.064)

^a^ All ORs were adjusted for age (continuous), race (Caucasian [reference], Black, American Natives, and other), and gender (male [reference], female). Shaded area indicates statistical significance at a level of 5%. ^b^ Best available reference for comparison.

**Table 5:** RMSE of ORs for integer rural-urban codes as compared to the continuous rurality index.

Error Statistic	RUCC-County	RUCA-ZCTA	RUCA-Tract
RMSE	0.029	0.030	0.021

## Appendix A

**Table A1:** Primary cancer site definitions by ICD-9 codes.

Primary Cancer Site	ICD-9 codes
Breast (female)	C500, C501, C502, C503, C504, C505, C506, C508, C509
Prostate	C619
Colorectal	C180, C181, C182, C183, C184, C185, C186, C187, C188, C189, C209, C260
Lung	C340, C341, C342, C343, C348, C349
Hematologic	C420, C421, C422, C770, C771, C772, C773, C774, C775, C778, C779

**Table A2:** Macro-components and factors to define the continuous rurality index [30].

Macro-Component	Factor	Definition	Literature
Demography	Population density	Residential population per unit of area	[39]
Socio-economy	Ethnic diversity	Number of different ethnic groups	[46]
	Land use	Degree of land development	[39]
	Income	Mean household income	[47]
Accessibility	Transportation network	Density of roads	[40]
	Access to healthcare	Density of health facilities	[40,48]
	Access to social service	Density of social service facilities	[48]
